# Short-Chain Fatty Acids Reduce Oligodendrocyte Precursor Cells Loss by Inhibiting the Activation of Astrocytes via the SGK1/IL-6 Signalling Pathway

**DOI:** 10.1007/s11064-022-03710-0

**Published:** 2022-09-13

**Authors:** Yanmin Gao, Di Xie, Yang Wang, Lei Niu, Hua Jiang

**Affiliations:** 1 Department of General Practice, Shanghai East Hospital, School of Medicine, Tongji University, No.150, Jimo Road, Pudong New District, Shanghai, 200120 China; 2 Emergency Department, Xinhua Hospital, Shanghai Jiao Tong University School of Medicine, No.1665, Kongjiang Road, Yangpu District, Shanghai, 200092 China; 3 Department of General Practice, Kongjiang Community Health Service Center, No. 100, Yanji West Road, Yangpu District, Shanghai, 200093 China

**Keywords:** SCFAs, IL-6, Astrocytes, Bax, SGK1

## Abstract

Short-chain fatty acids (SCFAs) are known to be actively involved in neurological diseases, but their roles in hypoxic-ischaemic brain injury (HIBI) are unclear. In this study, a rat model of HIBI was established, and this study measured the changes in IL-6 and NOD-like receptor thermal protein domain associated protein 3 (NLRP3), in addition to proliferation and apoptosis indicators of oligodendrocyte precursor cells (OPCs). The mechanism of action of SCFA on astrocytes was also investigated. Astrocytes were subjected to hypoxia in vitro, and OPCs were treated with IL-6. The results showed that SCFAs significantly alleviated HIBI-induced activation of astrocytes and loss of OPCs. SCFA pretreatment (1) downregulated the expression of NLRP3, IL-6, CCL2, and IP-10; (2) had no effect on the proliferation of OPCs; (3) ameliorated the abnormal expression of Bax and Bcl-2; and (4) regulated IL-6 expression via the SGK1-related pathway in astrocytes. Our findings revealed that SCFAs alleviated the loss of OPCs by regulating astrocyte activation through the SGK1/IL-6 signalling pathway.

## Introduction

Stroke is often associated with a poor neurological prognosis, and hypoxic-ischaemic brain injury (HIBI) is the main mechanism underlying this effect [1]. Axonal demyelination and neuroinflammation are important features of HIBI [2–4]. The adult brain contains a large population of oligodendrocyte progenitor cells (OPCs), which affect myelination and astrocyte-related neuroinflammation. Our previous study showed that HIBI results in axonal demyelination [3, 5–7], but the relationship between astrocytes and OPCs is unclear. Activation of astrocytes may be involved in OPC injury.

Recent studies have shown that activated astrocytes secrete inflammatory factors and chemokines that are involved in the regulation of neuroinflammation and that regulating astrocyte overactivation is a promising strategy for the treatment of HIBI [8, 9]. The NOD-like receptor thermal protein domain associated protein 3 (NLRP3) inflammasome is one of the best studied and characterized inflammasomes due to its unique response to a wide diversity of stimuli. NLRP3 has been suggested to play a role in a variety of disorders, and its over-activation may lead to severe impairments (i.e., cell swelling, tissue damage, internal bleeding and respiratory disablement) [10, 11]. These effects are mediated by caspase-1 activation and secretion of IL-1β, which ultimately triggers the pathophysiological changes of OPCs [3]. There is evidence that IL-1 leads to the increase of IL-6 in inflammatory states, and the latter cytokine may also be involved in NLRP3 mediated OPCS injury. However, the specific mechanism is not clear [12].

Recent studies have highlighted the important effects of natural products on brain diseases. Increasing evidence shows that short-chain fatty acids (SCFAs) are closely related to a variety of brain diseases. SCFAs, including acetate, propionate and butyrate, are produced by the metabolism of dietary fibre by intestinal flora. SCFAs can migrate from the intestine to the brain, play a variety of roles and regulate the function of the central nervous system [3, 13]. However, the mechanism by which SCFAs regulate neuroinflammation and OPC loss remains unclear and may involve astrocyte activation. Serum and glucocorticoid-induced protein kinase 1 (SGK1) is a protein kinase that is involved in cell metabolism [14], but the role of SGK1 in astrocyte activation is not clear. Whether SCFAs increase the activation of astrocytes during HIBI through the regulation of the SGK1 signalling pathway is unclear. The present study aims to explore the molecular mechanism of astrocyte activation after treatment with SCFAs in the context of HIBI and to provide a scientific theoretical basis for the treatment of HIBI.

## Materials and Methods

### Animals and Study Design

The animals were purchased from Jihui Experimental Animal Breeding Co., Ltd. (Shanghai, China). A total of 100 male SD rats weighing 200–250 g were selected. Pentobarbital (30 mg/kg) was injected intraperitoneally, and anaesthesia induction time was 20 min. In this study, animals were divided into the following groups: (1) the sham operation group, (2) the bilateral common carotid artery occlusion (BCCAO) group (two vessel occlusion, 2VO; data collection was stopped if an animal died prematurely), and (3) the SCFA (3:1:1 acetate: propionate: butyrate, 500 mg/kg, intragastric administration for 7 d before BCCAO) + BCCAO group. The rats were fed under normal oxygen conditions for 1, 3 and 7 d before sacrifice. The animals were euthanized via cervical dislocation. The animal treatment and experimental protocols were approved by the Shanghai Municipal Commission for Animal Protection and Use.

To assess the effect of SCFAs (lentivirus injection via the caudal vein) on astrocyte activation via SGK1, rats were divided into the sham operation group, BCCAO group, BCCAO + SCFA group, BCCAO + SCFA + SGK1 overexpression group, BCCAO + SCFA + si-SGK1 group, and SCFA group. The rats were fed under normal oxygen conditions for 1 d and then sacrificed.

### Primary Culture of Astrocytes and Oligodendrocytes (OLs)

Cells were cultured as described in the literature [3]. Briefly, mixed cells were isolated from 1-day-old SD rats. The cells were mixed for 10 d and then rotated at 180 RPM and 36.5 °C for 1 h to remove OPCs and microglia. The cells were maintained under normal conditions for 24 h and then subjected to different treatments. In this study, astrocytes with a purity of more than 90% were cultured. The purified OPCs were cultured in OPC medium (OPCM, proliferation medium) (ScienCell Research Laboratories, USA, Cat. No. 1601) for 1 d in 5% carbon dioxide (CO_2_) and 95% air at 37 °C.

### Treatment of OPCs and Astrocytes

The effect of SCFAs on OPCs under oxygen–glucose deprivation (OGD) conditions were explored. In the OGD study, glucose-deprived DMEM (GD-DMEM) was incubated in 1% oxygen 94% N_2_/5% CO_2_ in a tri-gas incubator (Astec, Japan) overnight to obtain OGD-DMEM. The next day, the cells were rinsed with GD-DMEM rather than OPCM and incubated with OPCs in OGD-DMEM for 1 d [15].

The cells were divided into the following groups: the control group, OGD group, and OGD + 40 nmol/L SCFA group.

To explore the effect of IL-6 on OLs, the following groups were used: the control group, 20 ng/ml IL-6 group, 20 ng/ml IL-6 + 20 ng/ml IL-6 antagonist (IL-6Ra) (sarilumab) group, and 20 ng/ml IL-6Ra group.

To determine the effect of SCFAs on NLRP3, IL-6, CCL2, and IP-10 levels in astrocytes, astrocytes were divided into the following groups: the control group, the OGD group, the OGD + 40 nmol/L SCFA group, and the 40 nmol/L SCFA group. In the OGD study, GD-DMEM was incubated in 1% oxygen 94% N2/5% CO2 in a tri-gas incubator (Astec, Japan) overnight to generate OGD-DMEM. The next day, astrocytes were rinsed with GD-DMEM and then incubated in OGD-DMEM for 1 h [1].

To assess the effect of SCFAs on SGK1 levels in astrocytes, cells were divided into the following groups: the control group, OGD group, OGD + 40 nmol/L SCFA group, OGD + 40 nmol/L SCFA + SGK1 group, and OGD + 40 nmol/L SCFA + si-SGK1 group (the transfection method is described in Ref. [6]).

To analyze whether SCFAs affect IL-6 levels via the SGK1 signalling pathway, cells were divided into the following groups: the control group, OGD group, OGD + 40 nmol/L SCFA group, OGD + 40 nmol/L SCFA + SGK1 group, OGD + 40 nmol/L SCFA + si-SGK1 group, and SCFA group.

### In Vivo Lentivirus Transfection

A U6-sh-SGK1-CMV-GFP lentiviral vector, which was used to silence SGK1 expression and a U6-overexpression-SGK1-CMV-GFP lentiviral vector, which was used to increase SGK1 expression, were constructed by GeneChem (Shanghai, China). The titres were 1 × 10^9^ TU/ml, and 10 µl lentivirus was injected into rats in the appropriate groups via the caudal vein.

### Plasmid Transfection

The SGK1 overexpression gene or sh-SGK1 was introduced into the pEGFP vector to silence the SGK1 gene after OGD exposure. Plasmids containing 4 µg DNA were dissolved in 240 µL of serum-free Opti-MEM. A total of 10 µL Lipofectamine 2000 (Life, 11,668,027) was dissolved in 240 µL serum-free Opti-MEM mixed with the plasmid solution in a tube for 20 min at room temperature. Astrocytes were cultured in a six-well plate and incubated under OGD conditions for 1 h. Western blot analysis was then carried out as described below.

### Western Blotting

Proteins were extracted from cerebral cortex tissues or primary astrocytes using a protein extraction kit (Pierce Biotechnology Inc., IL) according to the manufacturer’s protocol. The protein concentration in the supernatants was determined by a BCA Protein Assay Kit (Beyotime Biotechnology). Supernatant samples containing 50 μg of protein were heated to 95 °C for 10 min and separated by sodium dodecyl sulfate–polyacrylamide gel electrophoresis on a 10% gel in a Mini-Protein three apparatus (Bio-Rad Laboratories, Hercules, CA). The proteins were electroblotted onto 0.45 μm polyvinylidene difluoride membranes (Bio-Rad) at 1.5 mA/cm^2^ of membrane for 1 h in Towbin buffer (pH 8.3) mixed with 20% methanol [volume/volume (v/v)]. After transfer, the membranes were blocked with 5% (mass/vol) non-fat dried milk in Tris-buffered saline containing 0.05% Tween 20 (TBST) [0.05% (v/v) Tween-20 in 20 mmol/L (mm) Tris–HCl buffer (pH 7.6) containing 137 mm sodium chloride] for 1 h and then incubated with primary antibodies according to the manufacturer’s recommendations. IL-6 protein levels were determined by western blotting as described previously [3]. The following primary antibodies were used: IL-6 (1:1,000, Bosterm, China), NLRP3 (1:1,000, CST; USA), IL-6 receptor (IL-6R) (1:1,000, Santa Cruz, USA), Bax (1:1,000, Santa Cruz, USA); Bcl-2 (1:1,000, Santa Cruz, USA), PCNA (1:1,000, Santa Cruz, USA), Ki-67 (1:1,000, Santa Cruz, USA), GFAP (1:1,000), CCL2 (1:1,000), IP-10 (1:1,000) and β-actin (1:1,000) (all from Cell Signalling Technology). After washing with TBST three times, the cell membrane was incubated with HRP for 1 h. Then, immunoluminescence was performed.

### Double Immunofluorescence

Samples from each group were incubated with antibodies against IL-6 (1:100) and GFAP (1:100) and IL-6R (1:100). To assess the apoptosis and proliferation of OPCs, cells were incubated with anti-cleaved caspase-3 (1:100) and anti-BrdU (1:100) antibodies as described previously [6]. A total of eight randomly selected microscopic fields (each with area of 4.5 mm^2^) from each section were analyzed at 40 ×  Cells with an NG2-positive (green) body and a cleaved Caspase-3 (red)- or BrdU (red)-positive nucleus were counted as apoptotic or proliferating OPCs, and the number of apoptotic or proliferating OPCs was divided by the total number of NG2-positive cells. Cells that emitted green fluorescence only were counted as nonapoptotic or nonproliferative OPCs. The total number and percentage of apoptotic and proliferative OPCs were calculated and averaged. Cells were incubated with an anti-NG2 or anti-PDGFR-α (1:100) antibody overnight at 4 °C followed by secondary antibody and observed under a fluoroscope. The cells were then incubated with an Alexa Fluor 555-conjugated goat anti-rabbit secondary antibody (1:100, Life, Cat. No. A-21428) and FITC-conjugated goat anti-mouse secondary antibody (1:100 Chemicon International, Cat. No. AP130F) for 1 h. Finally, the cells were counterstained with DAPI and examined under a fluorescence microscope (Olympus System Microscope Model BX53, Olympus Company Pte, Tokyo, Japan).

### Electron Microscopy

The animals were perfused with 0.1 M phosphate buffer containing a mixture of 2% paraformaldehyde and 3% glutaraldehyde. Then, the brains were removed, and coronal sections (approximately 1 mm thick) were cut. The sections were then fixed in 1% osmium tetroxide for 2 h, dehydrated, and subsequently processed. Ultrathin sections were cut and observed under an electron microscope (FEI Corporation, Hillsboro, OR).

### Statistical Analysis

All data were evaluated using SPSS 13.0. The data are expressed as the mean ± standard deviation. Univariate data with homogeneity of variance were compared between four groups by analysis of variance (ANOVA). In addition, Welch’s ANOVA was used for analysis. If the data had homogeneity of variance, the least significant difference (LSD) method was used for multiple comparisons. P < 0.05 was considered statistically significant.

## Results

### IL-6 and NLRP3 Expression Levels In Vivo

**Fig. 1:**
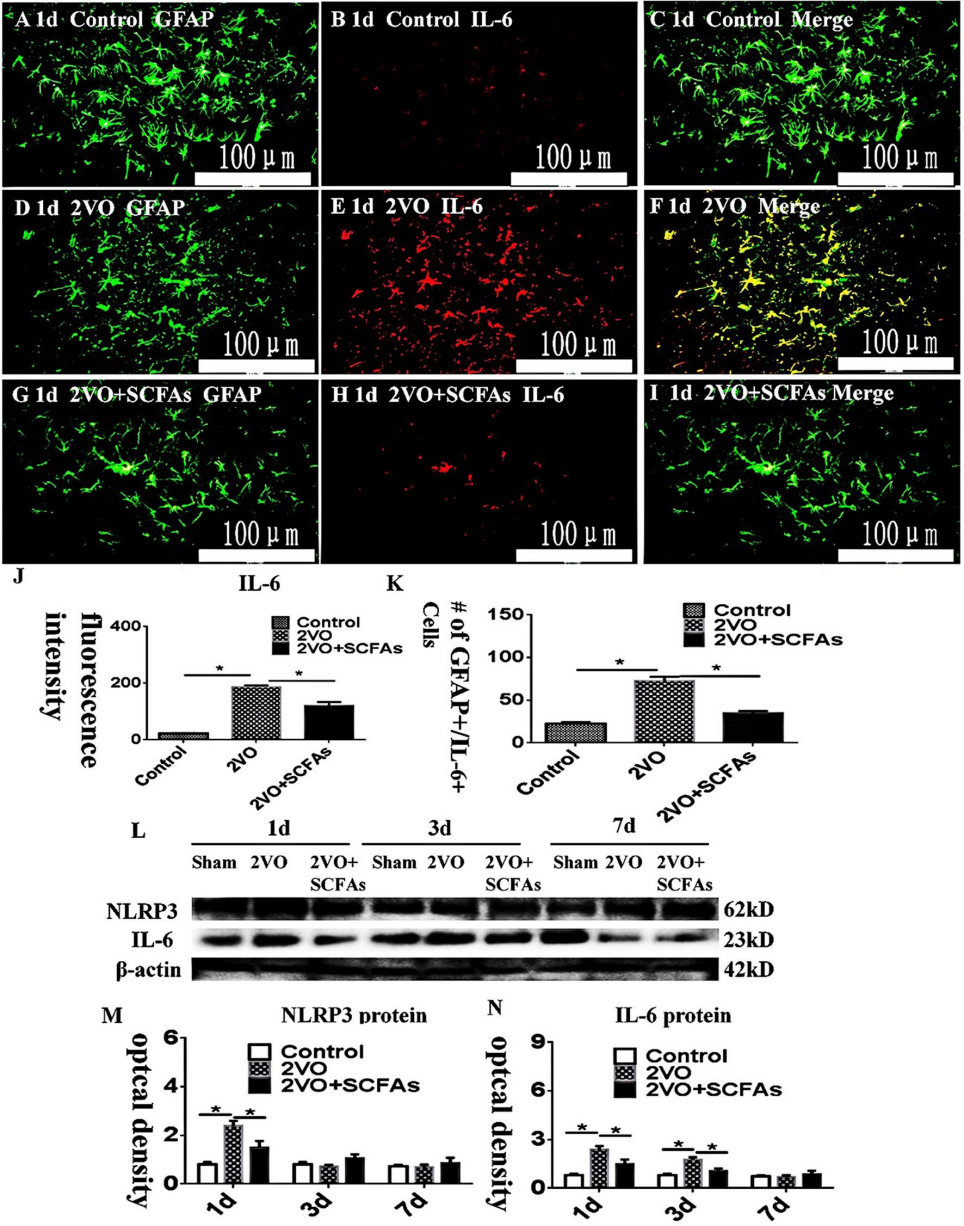
**A**–**M** Double immunofluorescence staining showing the distribution of GFAP- (green) and IL-6- (red) labeled immunoreactive astrocytes in vivo. **C**, **F**, and **I**, **J** the number of positive cells showed in K.Colocalized expression of GFAP and IL-6 in astrocytes. **L** NLRP3, IL-6 , and β-actin bands. **M** and **N** compared to control group, NLRP3 and IL-6 increased significantly after BCCAO. However, SCFAs can downregulate the expression of NLRP3 and IL-6 at 1 day. *N*=5 experiments were performed in independent preparations. *P < 0.05. Scale bars: A–I 100 μm.

In the corpus callosum (CC), IL-6 expression levels were measured (Fig. 1A–J). IL-6 immunoreactivity was higher in the BCCAO groups (Fig. 1D–F) than in the control group (Fig. 1A–C). However, SCFAs decreased the expression of IL-6 (Fig. 1G–I). The number of positive cells is shown in K. The immunoreactive bands of NLRP3 and IL-6, which appeared at approximately 62 and 23 kDa, respectively (Fig. 1L), increased significantly in optical density at 1 day after BCCAO when compared with the controls. This effect was significantly inhibited in the presence of SCFAs (Fig. 1)M and N.

### OPC Apoptosis in the CC

**Fig. 2:**
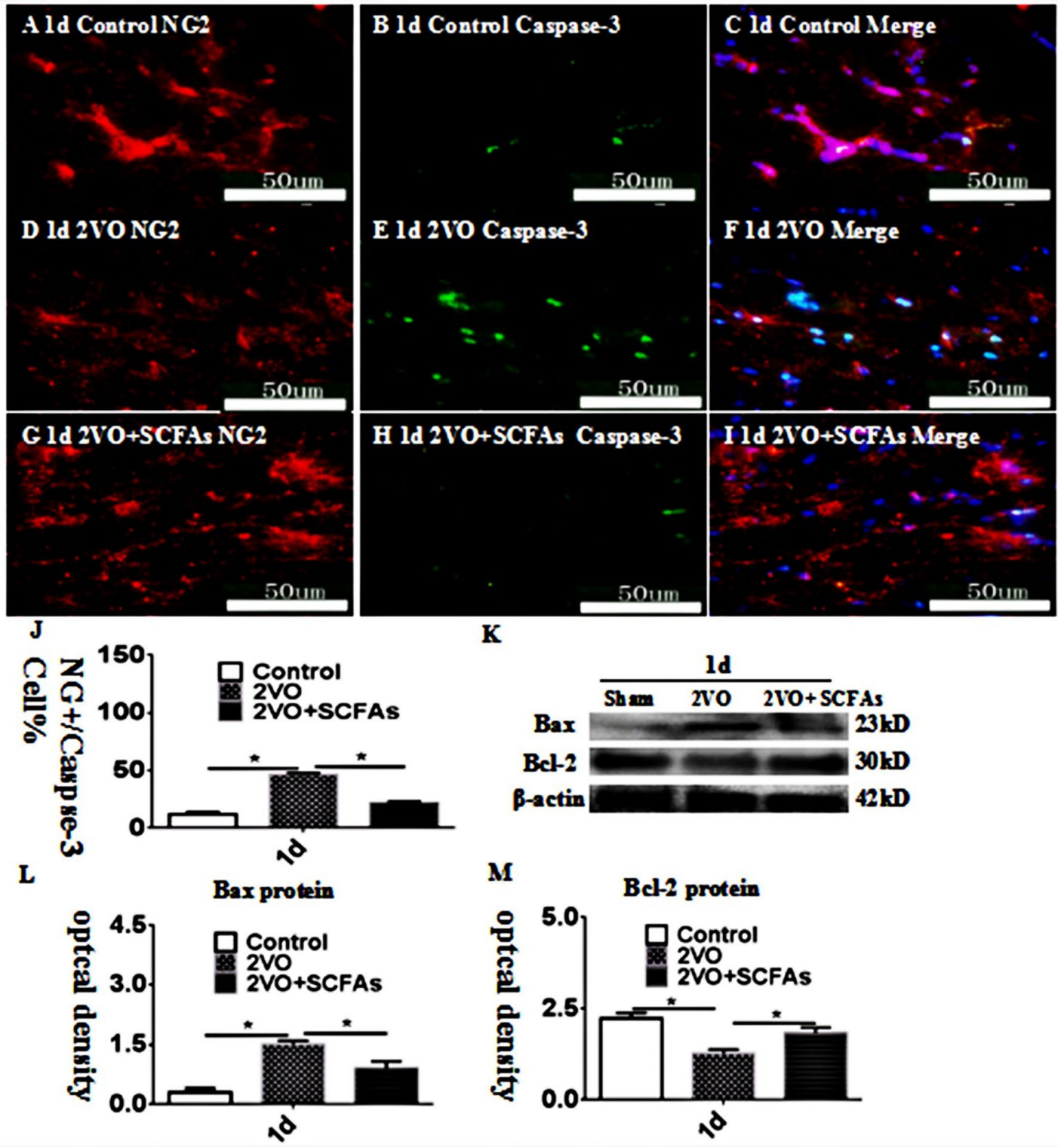
Immunofluorescence staining showing cleaved-caspase-3 immunoreactive cells (green) in the CC of rats (**A**–**I**). Bar graph in J shows a increase in the CC at 1 day after the BCCAO; SCFAs could improve the apoptosis. Panel **K**–**M** shows Bax and Bcl-2 immunoreactive bands. *N* = 5. experiments were performed in independent preparations. *P < 0.05. Scale bars: A–I 50 μm

After BCCAO, an increase in cleaved caspase-3 levels was observed (Fig. 2A–J). Cleaved caspase-3 levels in the CC were increased in the BCCAO group (Fig. 2D–F) compared to the control group (2A-2C) 1 d after surgery. These changes were reversed by SCFA treatment (Fig. 2G–J). The observed trend in Bax immunoreactivity was identical to that revealed by double immunofluorescence (Fig. 2K–L), and the trend in the expression of bcl-2 was opposite that in the expression of Bax (Fig. 2K–M).

### OPC Proliferation in the Cc

**Fig. 3:**
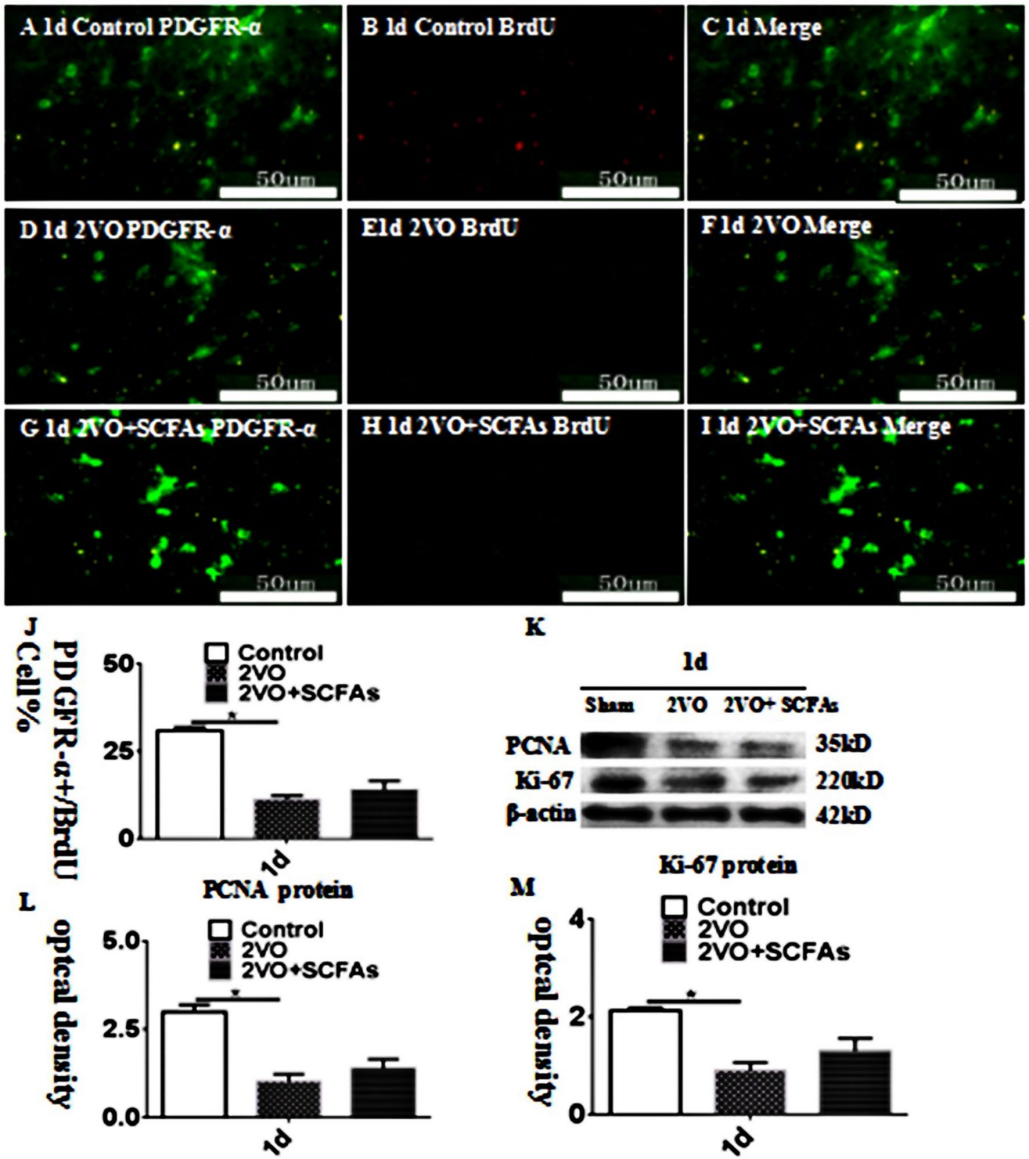
**A**–**J** show PDGFR-α and BrdU positive cells expressionlevel in vivo at 1 days. PDGFR-α (green) and BrdU (red). Panels **K**–**M** show bar graphs depicting significant decreases in PCNA and Ki-67 after BCCAO when compared to controls. SCFAs could improvement this. *N* = 5. experiments were performed in independent preparations. *P < 0.05. Scale bars: A–I 50 μm

The number of PDGFR-α + OPCs was significantly decreased in the BCCAO group (Fig. 3D–F) compared with the control group (Fig. 3A–C). However, SCFAs had no effect on the number of BrdU + OPCs (Fig. 3G–J). The observed trends in the protein expression of PCNA and Ki-67 were identical those revealed by double immunofluorescence, and SCFAs had no effect on the levels of these proteins (Fig. 3K–M). The experimental data showed that SCFAs had no effect on the proliferation of OPCs. SCFAs may affect the number of OPCs in other ways.

### SCFAs Affect the Expression of SGK1 and GFAP and OPC Apoptosis

**Fig. 4:**
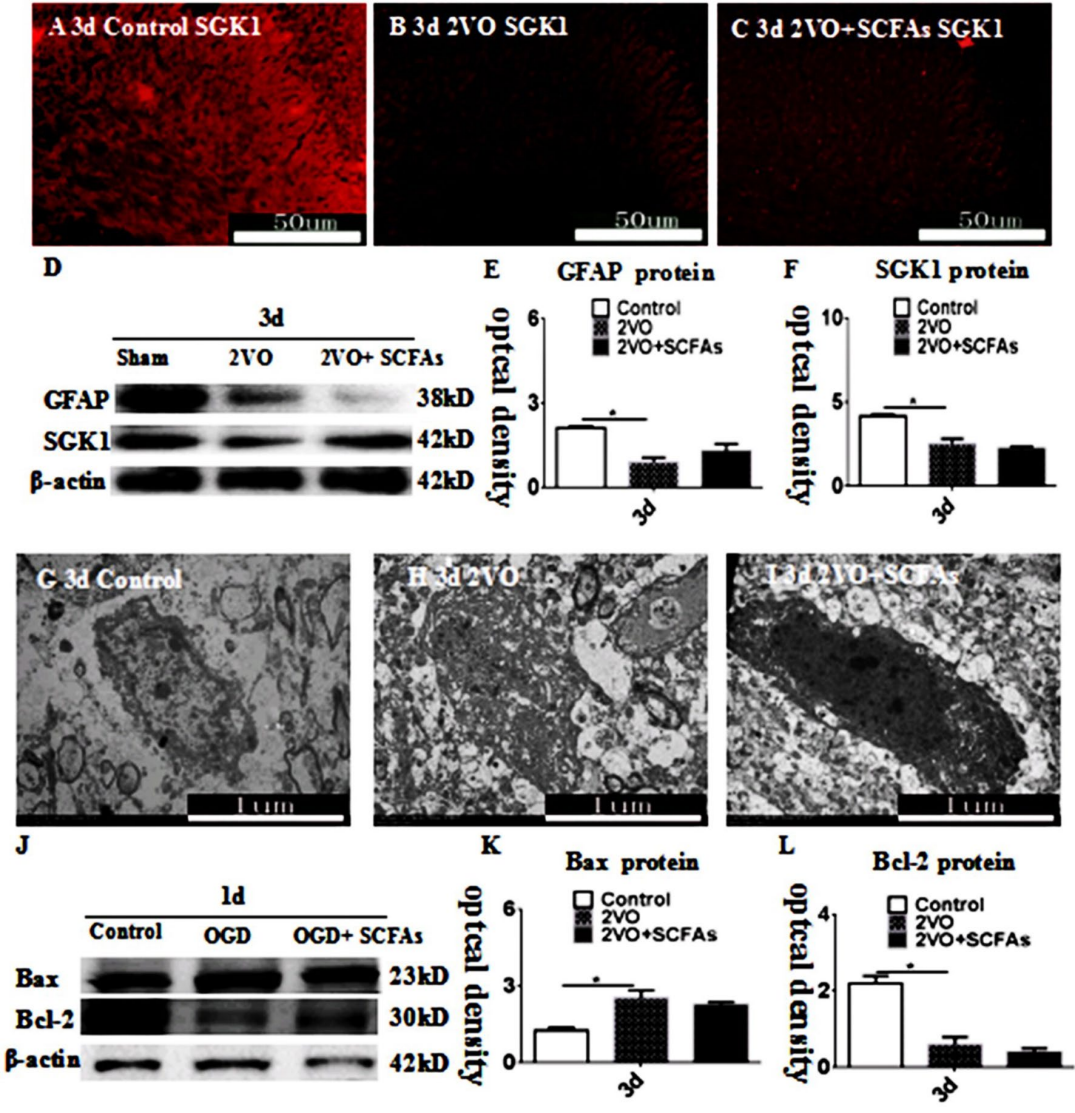
SCFAs improves expression of SGK1 protein in CC. SGK1 (red) immunoreactive in CC at 3d (**A**–**C**). Bar graph **D**–**F** depicting significant decrease expression of GFAP and SGK1 expression. **H** showed nuclear fragmentation in OPCs compared to controls (**G**), SCFAs could improvement this (**I**). SCFAs had no effect on the apoptosis of OPCs by itself (**J**–**L**), *N* = 5. experiments were performed in independent preparations. *P < 0.05. Scale bars: A–C 50 μm. EM 1 μm

SGK1 immunoreactivity in the CC was decreased in the BCCAO group (Fig. 4B) compared with the control group (Fig. 4A). These changes were reversed by SCFA treatment (Fig. 4C). The observed trends in the expression of GFAP and SGK1 were identical to those revealed by double immunofluorescence, and SCFAs had no effect on GFAP expression (Fig. 4D–F). We used electron microscopy to observe nuclear fragmentation in OPCs. Nuclear breakage occurred in the BCCAO group compared with the control group (Fig.4 G–I). SCFAs decreased SGK1 levels and reduced OPC loss, and in vitro experiments showed that SCFAs alone had no effect on OPC apoptosis (Fig. 4J–L). These results suggest that SCFAs affect the loss of OPCs in other ways and that astrocyte-derived IL-6 may be involved in this effect.

### Expression of IL-6R in OPCs In Vivo

**Fig. 5:**
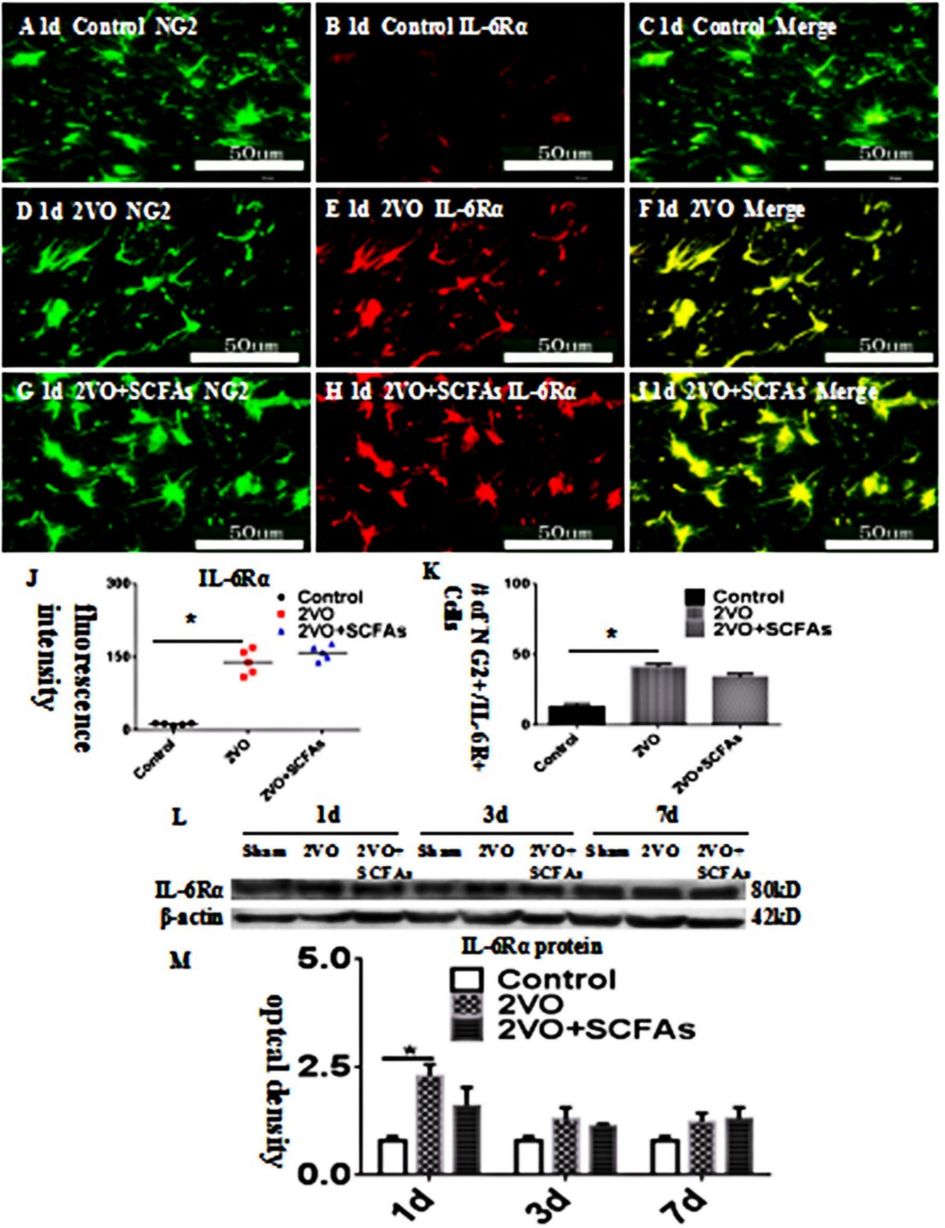
**A**–**J** IL-6 receptor α (IL-6Rα) expression in the corpus callosum. NG2- (green) and IL-6Rα- (red) labelled immunoreactive oligodendrocyte precursor cells (OPCs) in corpus callosum. The number of positive cells showed in (**K**). Panel **L** shows IL-6Rα bands. Panel **M** show bar graph depicting the expression of IL-6Rα following the BCCAO when compared to their controls. N = 5. experiments were performed in independent preparations. *P < 0.05. Scale bars: A–I 50 μm

IL-6R was localized to OPCs and was colocalized with NG2 (Fig. 5A–J). IL-6R immunoreactivity in OPCs was increased in the BCCAO group (Fig. 5D–F) compared with the control group (Fig. 5A–C). SCFAs had no effect on IL-6Ra levels (Fig. 5G–I). The number of IL-6Ra-positive cells is shown in Fig. 5K. The observed trend in the IL-6R protein level was identical to that revealed by double immunofluorescence (Fig. 5L–M).

### IL-6 Regulates OPC Apoptosis In Vitro

**Fig. 6:**
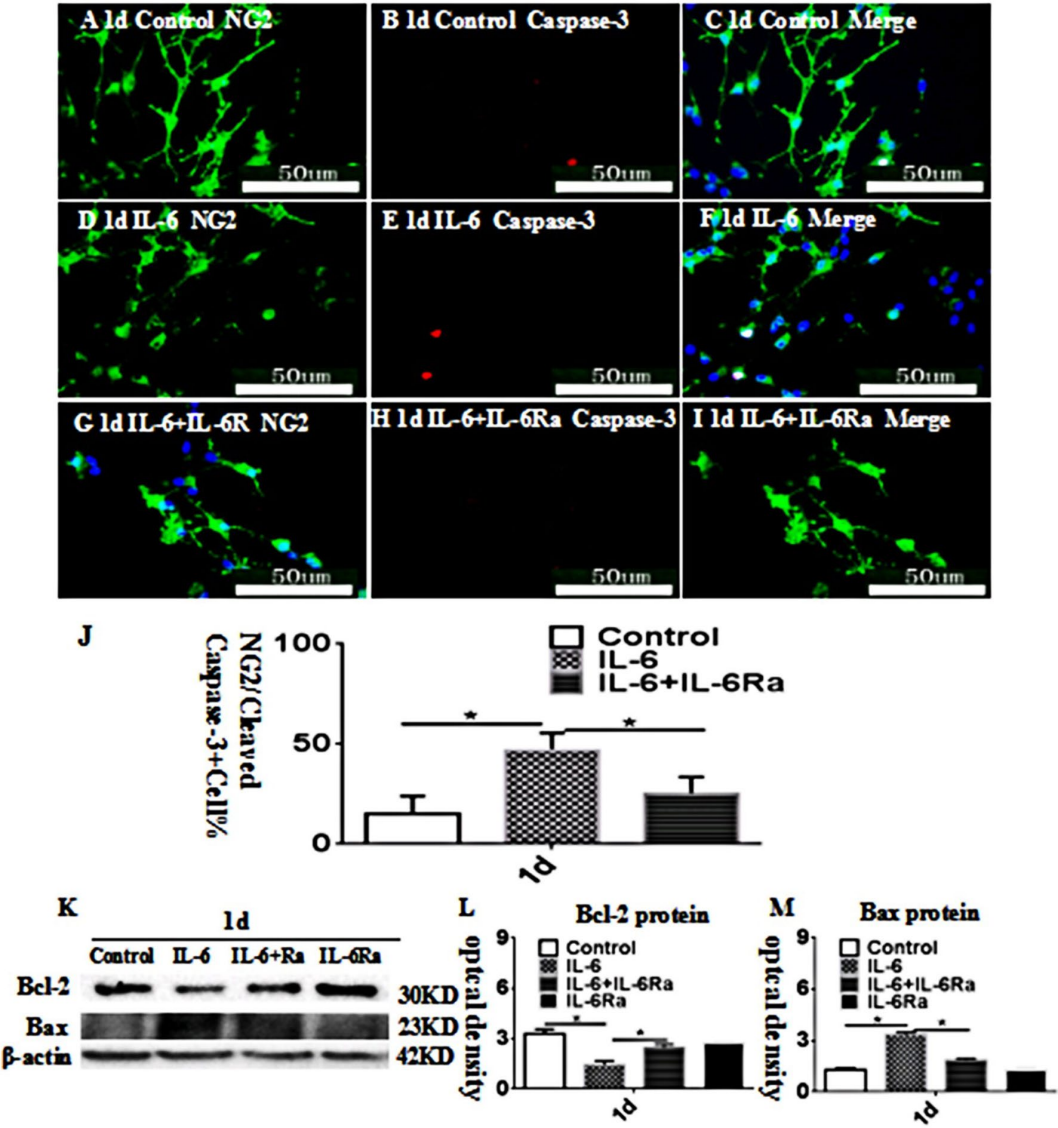
IL-6 promotes the apoptosis of OPCs in vitro. Immunofluorescence images of cultured OPCs show the expression of NG2 (**A**, **D**, **G**, green), caspase-3 (**B**, **E**, **H**, red). The co-localized expression of NG2 and caspase-3 can be seen in panels **C**, **F**, **I**. Bar graph in **J** shows the number of caspase-3/NG2-positive OPCs/mm2 at 1 d after the IL-6 administration or IL-6+IL-6R atreatment when compared with the corresponding control. Note that IL-6Ra attenuates the apoptosis of OPCs and IL-6Ra may reverse the course. Panel **K** shows Bcl-2 (30 kDa), Bax (23 kDa), and â-actin (42 kDa) immunoreactive bands. Bar graphs in **L** and **M** show significant decreases or increases in the optical density of Bcl-2 or Bax at 1 d after IL-6 administration when compared with the corresponding controls. The IL-6Ra antagonist may attenuate the increment

Double immunofluorescence showed that very few OPCs were double labeled with NG2 and caspase-3 at 1 day after treatment with IL-6 or IL-6+IL-6Ra and the matching controls (Fig. 6A-I). There were more NG2-labeled OPCs that were caspase-3 immunoreactive at 1 d aftertreatment with IL-6 (Fig. 6D-F) in comparison with the control (Fig.6A-C). However, IL-6Ra significantly reversed the caspase-3 approximately 23KD and 30KD, respectively (Figs. 6K), decreased (Bcl-2) or increased (Bax) significantly in optical density at 1 d after after treatment with IL-6 as compared with the controls (Figs. 6L, M). However, IL-6Ra significantly improve the above phenomena (Figs. 6L, M). These data demonstrated that the apoptosis of OPCs was significantly activated after treatment with IL-6 as compared with the controls.

### SCFAs Can Inhibit IL-6 Expression In Vitro

**Fig. 7:**
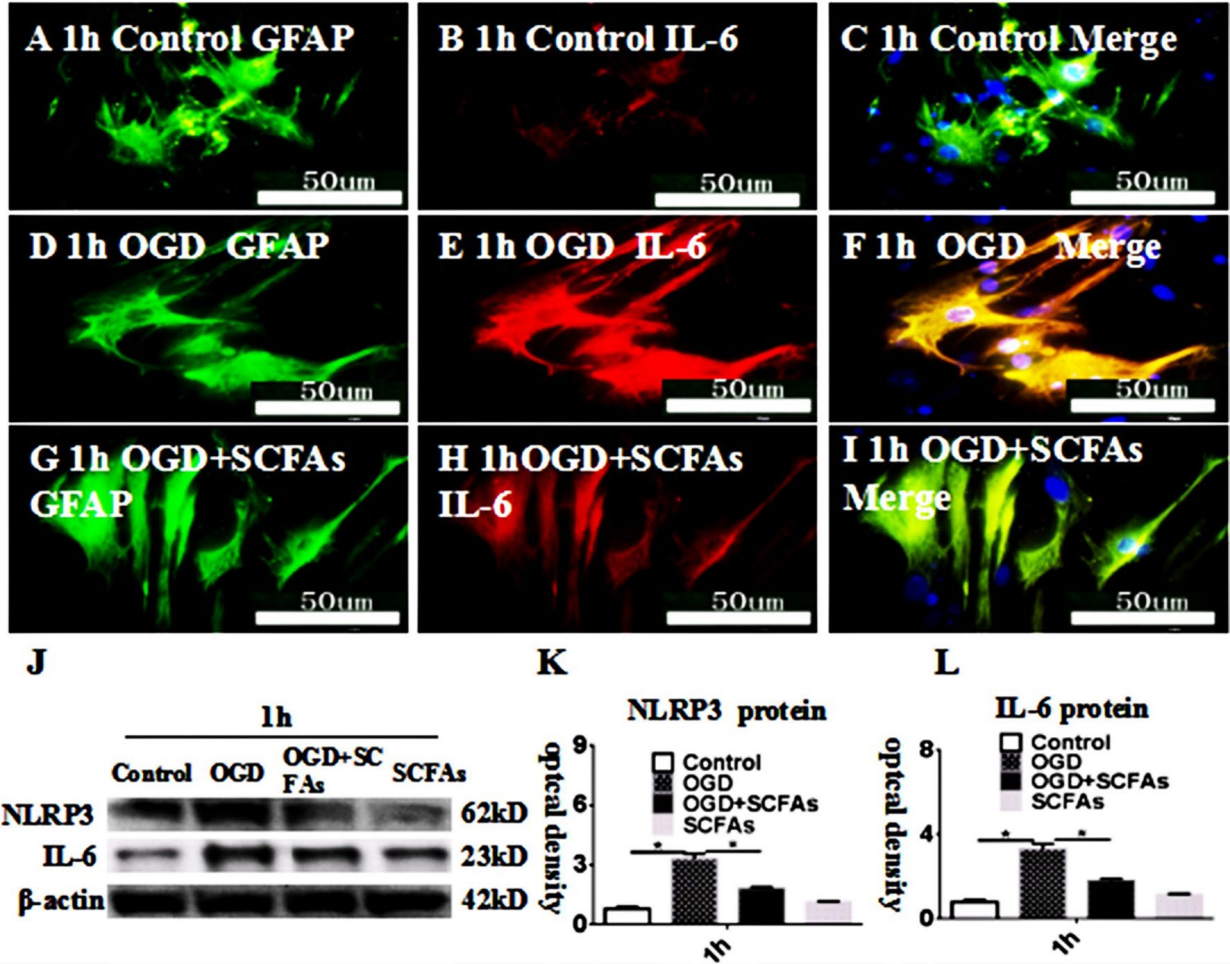
SCFAs inhibited the expression of IL-6 and NLRP3 in astrocytes after OGD in vitro. Immunofluorescence images show the expression of GFAP (**A**, **D**, **G**, green), IL-6 (**B**, **E**, **H**, red). The co-localized expression of GFAP and IL-6 can be seen in panels **C**, **F**, **I**. Panel **J** shows NLRP3 (62 kDa), IL-6 (23 kDa), and â-actin (42 kDa) immunoreactive bands. Bar graphs in **K** and **L** show significant increases in the optical density of NLRP3 and IL-6 at 1 h after OGD administration when compared with the corresponding controls. The SCFAs antagonist may attenuate the increment

Double immunofluorescence showed that IL-6 was increased at 1 h after treatment with OGD (Fig. 7D-F) in comparison with the control (Fig. 7A-C). However, SCFAs significantly reversed the IL-6 immunoreactivity in astrocytes as treated with OGD (Fig. 7G-I). The immunoreactive bands of NLRP3 and IL-6 protein levels that appeared at approximately 62 kDa,23 kDa, respectively (Fig. 7J), increased significantly in optical density at 1 h after after treatment with OGD as compared with the controls. However, SCFAs significantly improve the above phenomena (Fig. 7K, l). These data demonstrated that the expression of IL-6 and NLRP3 was significantly inhibited after treatment with SCFAs as compared with the OGD.

### SCFAs Regulate CCL2 and IP-10 Levels in Astrocytes

**Fig. 8:**
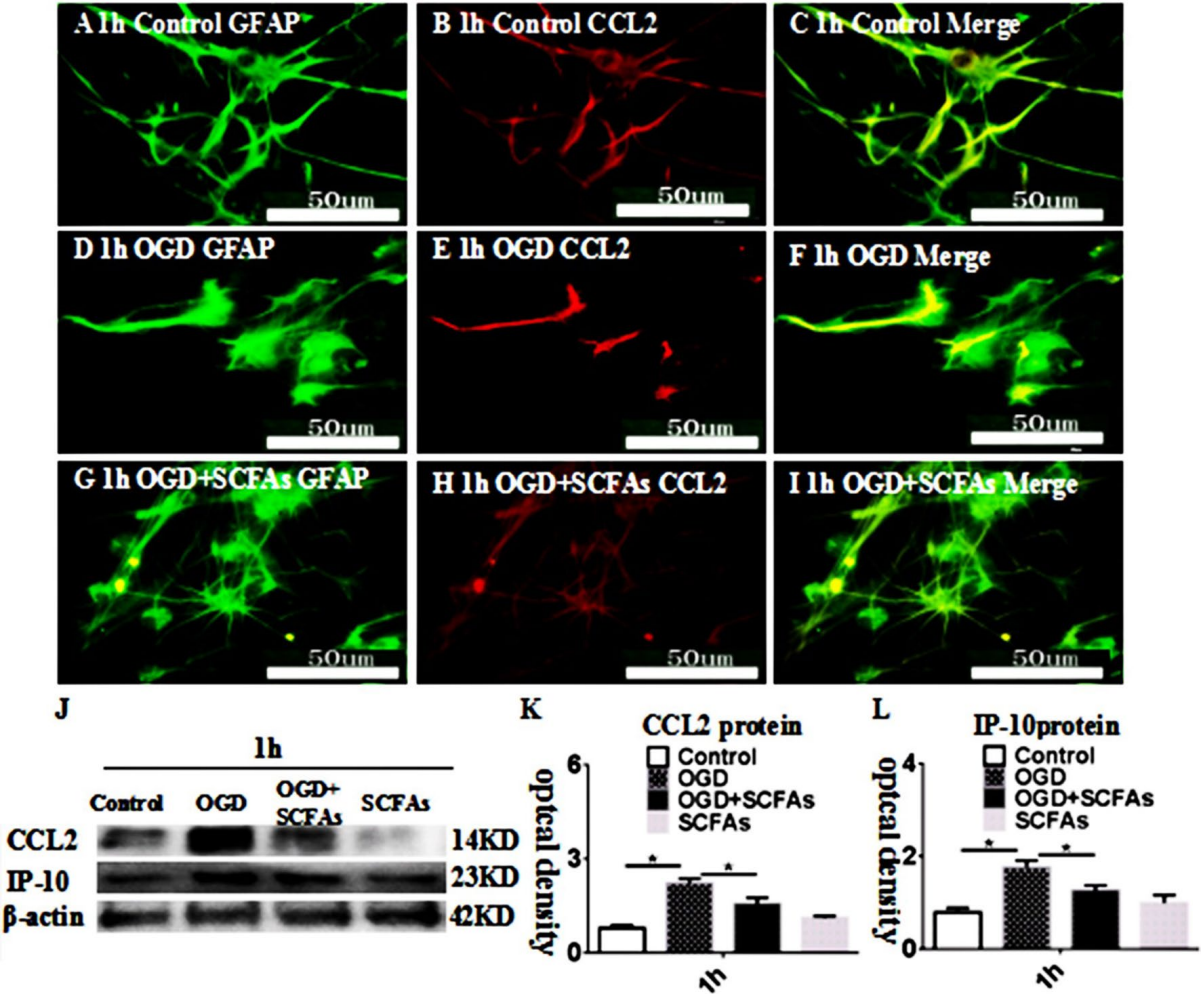
SCFAs regulates endoplasmic reticulum stress in astrocytes in vitro. Immunofluorescence images show the expression of GFAP (**A**, **D**, **G**, green), CCL2 (**B**, **E**, **H**, red). The co-localized expression of GFAP and CCL2 can be seen in panels **C**, **F**, **I**. Panel **J** shows CCL2 (14 kDa), IP-10 (23 kDa), and â-actin (42 kDa) immunoreactive bands. Bar graphs in **K** and show significant increases in the optical density of CCL2 and IP-10 at 1 h after OGD administration when compared with the corresponding controls. The SCFAs antagonist may attenuate the increment. *P < 0.05. Scale bars: A–I 50 μm

CCL2 expression was upregulated in the OGD group compared with the control group at 1 h. These changes were reversed by SCFA treatment (Fig. 8G–I). The levels of CCL2 and IP-10 were increased in the OGD group compared with the control group at 1 h (Fig. 8J–L). SCFAs alleviated the overexpression of the CCL2 and IP-10 proteins. The above results show that SCFAs regulate astrocyte activation in vitro; however, the mechanism is unclear.

### SCFAs Regulate IL-6 Expression via SGK1 In Vivo and In Vitro

**Fig. 9:**
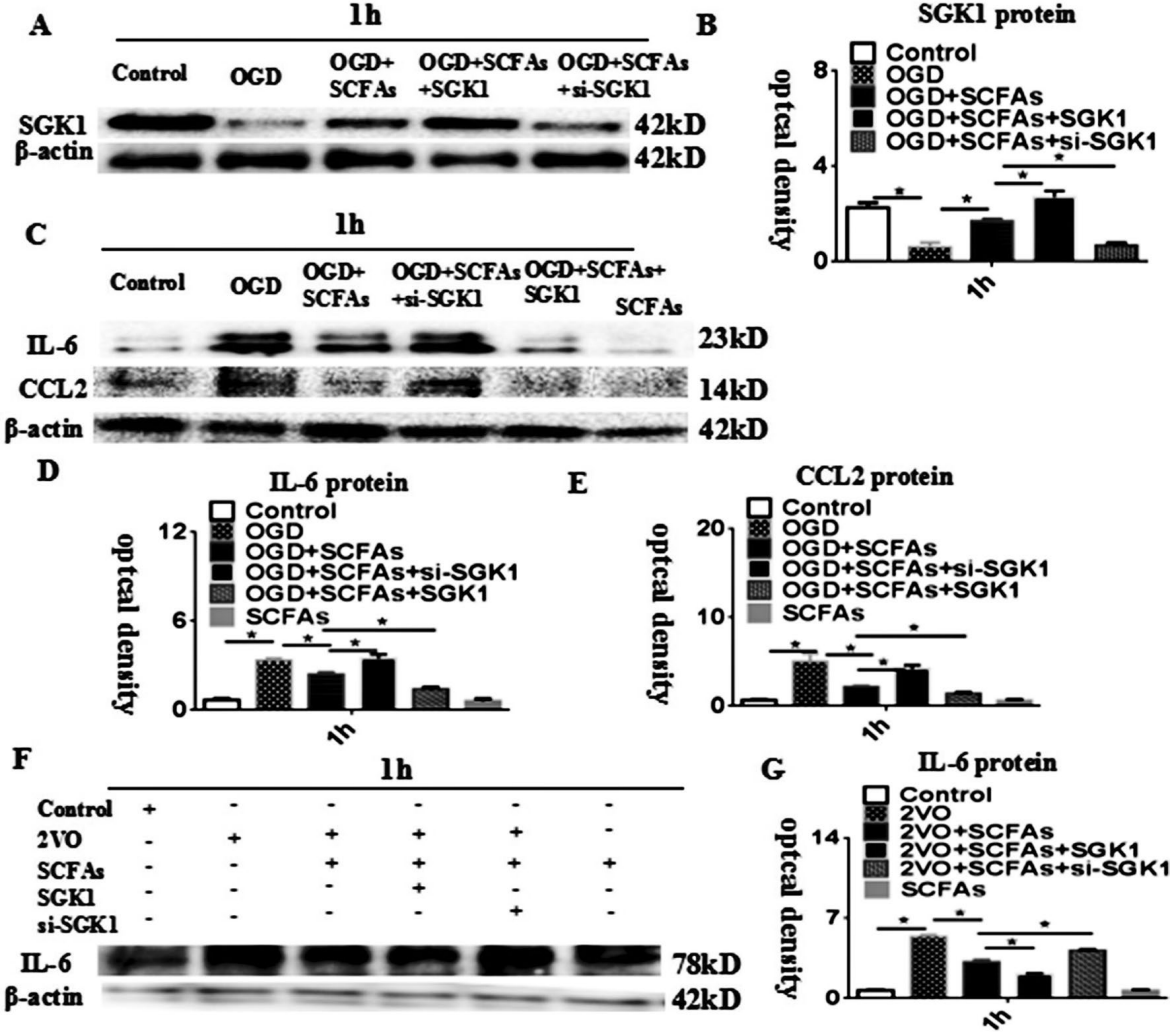
SCFAs regulates IL-6 via SGK1 in astrocyte. **A** Western blot of protein expression levels of SGK1 in astrocytes at 1 h (42 kDa). **B** Significantly downregulated protein expression level of SGK1 at after OGD. **C** IL-6 and CCL2 levels in astrocytes at 1 h. **D**–**E**) Significantly upregulated protein level of IL-6 and CCL2 at 1 h after treatment with OGD compared to control group. overexpression-SGK1 can improve IL-6 and CCL2 proteins. The effect weakens after adding si-SGK1. (**G**) Significantly downregulated protein expression level of IL-6 after treatment with SGK1 gene overexpression after OGD + SCFAs compared with that of the OGD + SCFAs. N = 5 experiments were performed in independent preparations. *P < 0.05

We hypothesized that SCFAs regulate IL-6 expression via the SGK1 signalling pathway. SGK1 expression was markedly downregulated in the OGD group compared with the control group at 1 h. SCFAs increased SGK1 protein levels, and this effect was weakened by treatment with SGK1 siRNA. When SGK1 was overexpressed, the opposite result was obtained (Fig. 9A–B). The protein level of IL-6 was markedly increased in the OGD group compared with the control group. Overexpression of the SGK1 gene alleviated the overexpression of the IL-6 and CCL2 proteins. si-SGK1 transfected had the opposite effect (Fig. 9C–E). To further verify the regulatory effect of SGK1 on IL-6 expression, in vivo experiments were performed. The results showed that the effect of SCFAs was weakened after transfection of si-SGK1. However, SGK1 overexpression had the opposite effect (Fig. 9F and G). Therefore, the in vivo and in vitro data confirmed the effectiveness of SCFAs in reducing astrocyte activation via the SGK1/IL-6 signalling pathway.

## Discussion

Excessive inflammatory factor release is a sign of astrocyte activation [16]. SCFAs are known to be actively involved in neurological diseases, but their roles in HIBI are unclear [10]. The results showed that NLRP3 and IL-6 expression was upregulated in rats after BCCAO and that activated astrocytes were the source of IL-6. Our results showed that the expression levels of the IL-6R were increased by BCCAO, and IL-6R on OPCs and OPCs may be the target cells of IL-6. OPCs are NG2- or PDGFR-α-positive bipolar cells that sequentially differentiate into OLs [17–19]. OLs are myelinating cells that are present in the central nervous system throughout adulthood. OPCs differentiate into OLs, and loss of OPCs results in neurotransmission dysfunction [20–22]. The results showed that cleaved caspase-3 and bax were significantly increased and bcl-2 levels were decreased after HIBI, suggesting that OPC apoptosis occurs after HIBI and that SCFAs can ameliorate apoptosis. Our results showed that SCFAs did not affect the proliferation of OPCs at 1 d. GFAP is a marker of astrocytes. SCFAs did not affect GFAP levels. SCFAs may affect the function of astrocytes, but the mechanism is unknown. SGK1 is a member of the AGC subfamily of protein kinases and is involved in cell metabolism [22–25]. Our results showed that SCFAs increased the expression of SGK1, indicating that they may attenuate astrocyte overactivation via SGK1 and that IL-6 may be involved in this process. To verify these findings, in vitro experiments were carried out. The results preliminarily showed that SCFAs could regulate IL-6 and NLRP3 expression in vitro. NG2-positive cells are thought to be mainly OPCs [26], and apoptosis of OPCs is recognized as the main mechanism of OPC loss [27]. The in vitro results showed that the number of NG2-/cleaved caspase-3-positive cells was increased after IL-6 treatment. IL-6Ra prevented OPC loss, indicating that IL-6 can promote apoptosis of OPCs. Controlling the secretion of IL-6 is the key to reducing the loss of OPCs. Overexpression of chemokines is an indicator of astrocyte activation [28–30]. The results showed that SCFAs inhibited the overexpression of cytokines. We hypothesized that SCFAs inhibit astrocyte activation by promoting the SGK1 signalling pathway. This study found that SGK1 expression levels were decreased after OGD exposure and that SCFAs increased SGK1 levels after OGD exposure. IL-6 and CCL2 expression levels increased after OGD exposure, whereas SCFAs increased IL-6 and CCL2 expression. In addition, when siRNA-SGK1 was added, this effect was ameliorated, indicating that SCFAs regulate the expression of IL-6 and CCL2 through SGK1. However, when SGK1 was overexpressed, the effect of SCFAs was strengthened, suggesting that SCFAs inhibit IL-6 expression via SGK1. To further determine the underlying mechanism, we performed in vivo validation experiments and obtained the same results. Therefore, SCFAs alleviate the activation of astrocytes by regulating the SGK1/IL-6 signalling pathway.

In summary, activated astrocytes in the CC produce excess IL-6, which induces neuroinflammation and OPC loss. However, SCFAs decrease IL-6 levels. Further analysis revealed that SCFAs reduce IL-6 levels by regulating the SGK1 signalling pathway in astrocytes. Further research on this process will facilitate the development of effective therapeutic strategies for relieving neuroinflammation.

## Data Availability

The data can be provided if necessary.

## Abbreviations

HIBI: Hypoxic-ischaemic brain injury
BCCAO: Bilateral common carotid artery occlusion
OLs: Oligodendrocytes
OPCs: Oligodendrocyte progenitor cells
SCFAs: Short-chain fatty acid
OGD: Oxygen-glucose deprivation
PBS: Phosphate-buffered saline
FBS: Foetal bovine serum

## References

[1] Guardia CM, Paez PM, Pasquini LA (2016). Inhalation of growth factors and apo-transferrin to protect and repair the hypoxic-ischemic brain. Pharmacol Res.

[2] Caraganis A, Mulder M, Kempainen RR (2020). Share interobserver variability in the recognition of hypoxic-ischemic brain injury on computed tomography soon after out-of-hospital cardiac arrest. Neurocrit Care.

[3] Xie D, Ge X, Ma Y (2020). Clemastine improves hypomyelination in rats with hypoxic-ischemic brain injury by reducing microglia-derived IL-1β via P38 signaling pathway. J Neuroinflammation.

[4] Fernández-Castaeda A, Chappell MS, Rosen DA (2020). The active contribution of OPCs to neuroinflammation is mediated by LRP1. Acta Neuropathol.

[5] Franklin RJ (2002). Why does remyelination fail in multiple sclerosis?. Nat Rev Neurosci.

[6] Xie D, Shen F, He S (2016). IL-1beta induces hypomyelination in the periventricular white matter through inhibition of oligodendrocyte progenitor cell maturation via FYN/MEK/ERK signaling pathway in septic neonatal rats. Glia.

[7] Colombo E, Farina C (2016). Astrocytes: key regulators of neuroinflammation. Trends Immunol.

[8] Apolloni S, Fabbrizio P, Parisi C (2016). Clemastine confers neuroprotection and induces an anti-inflammatory phenotype in SOD1(G93A) mouse model of amyotrophic lateral sclerosis. Mol Neurobiol.

[9] Yang H, Wen Y, Zhang M (2020). Share MTORC1 coordinates the autophagy and apoptosis signaling in articular chondrocytes in osteoarthritic temporomandibular joint. Autophagy.

[10] Mangan M, Olhava E, Roush WR (2018). Targeting the NLRP3 inflammasome in inflammatory diseases. Nat Rev Drug Discov.

[11] Zhang H, Zahid A, Ismail H (2020). An overview of disease models for NLRP3 inflammasome over-activation. Expert Opin Drug Discov.

[12] Escárcega R, Lipinski M, García-Carrasco M (2018). Inflammation and atherosclerosis: cardiovascular evaluation in patients with autoimmune diseases. Autoimmun Rev.

[13] Frost G, Sleeth ML, Sahuri-Arisoylu M (2014). The short-chain fatty acid acetate reduces appetite via a central homeostatic mechanism. Nat Commun.

[14] Roberts AJ, Khom S, Bajo M (2019). Increased IL-6 expression in astrocytes is associated with emotionality, alterations in central amygdala GABAergic transmission, and excitability during alcohol withdrawal. Brain Behav Immun.

[15] Chen YJ, Hsu CC, Shiao YJ (2019). Anti-inflammatory effect of afatinib (an EGFR-TKI) on OGD-induced neuroinflammation. Sci Rep.

[16] Munoz FM, Patel PA, Gao X (2020). Reactive oxygen species play a role in P2X7 receptor-mediated IL-6 production in spinal astrocytes. Purinergic Signal.

[17] Cayre M, Courte` s S, Martineau F (2013). Netrin 1 contributes to vascular remodeling in the subventricular zone and promotes progenitor emigration after demyelination. Development.

[18] Fancy SP, Zhao C (2004). Franklin RJ. Increased expression of Nkx2.2 and Olig2 identifies reactive oligodendrocyte progenitor cells responding to demyelination in the adult CNS. Mol Cell Neurosci.

[19] Menn B, Garcia-Verdugo JM, Yaschine C (2006). Origin of oligodendrocytes in the subventricular zone of the adult brain. J Neurosci.

[20] El Waly B, Macchi M, Cayre M (2014). Oligodendrogenesis in the normal and pathological central nervous system. Front Neurosci.

[21] Dulamea AO (2017). Role of oligodendrocyte dysfunction in demyelination, remyelination and neurodegeneration in multiple sclerosis. Adv Exp Med Biol.

[22] Elbaz B, Popko B (2019). Molecular control of oligodendrocyte development. Trends Neurosci.

[23] Miyamoto Y, Torii T, Tanoue A (2016). VCAM1 acts in parallel with CD69 and is required for the initiation of oligodendrocyte myelination. Nat Commun.

[24] Kobayashi T, Deak M, Morrice N (1999). Characterization of the structure and regulation of two novel isoforms of serum-and glucocorticoid-induced protein kinase. Int J Dermatol.

[25] Liu W, Wang X, Liu Z (2017). SGK1 inhibition induces autophagy-dependent apoptosis via the mTOR-Foxo3a pathway. Br J Cancer.

[26] Li R, Zhang P, Zhang M (2020). The roles of neuron-NG2 glia synapses in promoting oligodendrocyte development and remyelination. Cell Tissue Res.

[27] Xing B, Brink LE, Maers K (2018). Conditional depletion of GSK3b protects oligodendrocytes from apoptosis and lessens demyelination in the acute cuprizone model. Glia.

[28] Hu H, Tian M, Ding C (2019). The C/EBP homologous protein (chop) transcription factor functions in endoplasmic reticulum stress-induced apoptosis and microbial infection. Front Immunol.

[29] Klymenko O, Huehn M, Wilhelm J (2019). Regulation and role of the ER stress transcription factor CHOP in alveolar epithelial type-II cells. J Mol Med (Berl).

[30] Wilson MR, Eyre TA, Martinez-Calle N (2020). Timing of high-dose methotrexate CNS prophylaxis in DLBCL: an analysis of toxicity and impact on R-CHOP delivery. Blood Adv.

